# Neurological Soft Signs and Psychopathology in Chronic Schizophrenia: A Cross-Sectional Study in Three Age Groups

**DOI:** 10.3389/fpsyt.2018.00098

**Published:** 2018-03-26

**Authors:** Christina J. Herold, Marc M. Lässer, Ulrich Wilhelm Seidl, Dusan Hirjak, Philipp A. Thomann, Johannes Schröder

**Affiliations:** ^1^Department of General Psychiatry, Section of Geriatric Psychiatry, University of Heidelberg, Heidelberg, Germany; ^2^Center for Mental Health, Klinikum Stuttgart, Stuttgart, Germany; ^3^Department of Psychiatry and Psychotherapy, Central Institute of Mental Health, Medical Faculty Mannheim, Heidelberg University, Mannheim, Germany; ^4^Department of General Psychiatry, Center of Psychosocial Medicine, University of Heidelberg, Heidelberg, Germany

**Keywords:** schizophrenia, neurological soft signs, psychopathology, age, chronicity

## Abstract

As established in a wealth of studies subtle motor and sensory neurological abnormalities or neurological soft signs (NSS) are frequently found in patients with schizophrenia at any stage of their illness. However, the potential impact of chronicity and age on NSS was scarcely investigated. Therefore, we assessed NSS in 90 patients with subchronic (*n* = 22) or chronic (*n* = 68) schizophrenia and in 60 healthy controls who were assigned to three age groups (18–29, 30–49, and +50 years). NSS were measured on the Heidelberg Scale, psychopathological symptoms including apathy were rated on established instruments. As demonstrated by analysis of variance, NSS scores in patients were significantly (*p* < 0.05) increased relative to healthy controls. Significant age effects arose in all NSS subscores, with older subjects scoring well above the younger ones. These age effects were more pronounced in patients than controls, indicating that NSS in chronic schizophrenia exceed age-associated changes. Moreover, the NSS scores in patients were significantly associated with duration of illness, thought disturbance, positive symptoms, and apathy. These results were confirmed after age/duration of illness and years of education were partialed out and *via* regression analyses. Our findings conform to the hypothesis that NSS are associated with chronicity of the disorder as indicated by the correlations of NSS with both, duration of illness and apathy. The correlations between NSS and positive symptoms/thought disturbance correspond to the fluctuation of positive symptoms during the course of the disorder. The significantly more pronounced age effects on NSS in patients may either point to ongoing cerebral changes or to a greater susceptibility of patients toward physiological age effects, which may be mediated among other factors by a lower cognitive reserve.

## Introduction

Neurological soft signs (NSS)—i.e., subtle motor and sensory deficits—are frequently found in patients with schizophrenia when compared with healthy controls and patients with other psychiatric conditions such as affective disorders [for a overview see Ref. (1, 2)]. As established in previous studies, NSS decrease in the course of acute psychosis paralleling psychopathological symptoms, but do not normalize to the level typically found in healthy controls (3, 4). These findings were confirmed and extended in a recent meta-analysis (5), which demonstrated that this decrease mainly applies to patients with a remitting course.

Little is known about NSS in the lifespan of patients with chronic schizophrenia. One may expect NSS scores to increase with persistence of psychopathological symptoms in chronic schizophrenia. This hypothesis is supported by Bachmann et al. (3), who found stable or increasing NSS scores to be associated with an unfavorable outcome in 39 first-episode patients investigated upon first hospitalization and after 14 months. Actually, the association between persisting NSS and chronicity was confirmed by Prikryl and colleagues (6) in 68 first-episode patients investigated over a follow-up period of 4 years. While NSS decreased in patients with a remitting course, scores increased in those prone to chronic schizophrenia. Similar results were reported in a previous study (7). Along with this, a study including 37 chronically hospitalized patients with schizophrenia (duration of illness: 22.0 years, SD = 9.1) found NSS to be rather stable during a 5-year period (8). Unfortunately, further longitudinal studies that investigated NSS over similar periods of time are not available (5).

While these findings indicate that NSS may be associated with progressive cerebral changes (9), NSS also share trait characteristics which may refer to an early congenital or neurodevelopmental assault. For discussion of this state-trait dichotomy, see Bachmann and Schröder in this volume (10).

When investigating chronicity, age has to be considered as a potential confounding factor. Recent studies foster the hypothesis of accelerated aging in schizophrenia (11) as patients show cognitive changes (12, 13) and physical diseases at a younger age than healthy controls (14, 15). This hypothesis corresponds to the lower life expectancy of patients, which is still reduced by up to 20 years [(16), see also Ref. (17)] and parallels Kraepelin’s concept of dementia praecox (18). In this context, apathy should also be considered as it is defined as a much broader concept than mere negative symptoms. Apathetic symptoms are not only found in patients with dementia but also in patients with chronic schizophrenia (19, 20).

We therefore sought to compare NSS in a large sample of patients with chronic schizophrenia and in healthy controls from different age groups to especially focus on the contributions of age and chronicity of the disorder on NSS. We expected NSS scores to increase with both chronicity of the disease and age, and that the interaction of both should differ between patients and controls.

## Materials and Methods

### Participants

Between 2008 and 2011, 90 patients with subchronic (*n* = 22) or chronic (*n* = 68) schizophrenia (*N* = 86) or schizoaffective disorder (*N* = 4) by DSM-III/DSM-IV criteria (21, 22) were recruited from three psychiatric long-term units (*n* = 37) and a mental state hospital (*n* = 53). Sixty healthy controls matched for age and sex were recruited through newspaper advertisement. Age of disease onset was determined on the basis of the patients’ history and case notes. Patients with late onset schizophrenia (23), i.e., with a manifestation of the disease after age 40 or severe extrapyramidal symptoms were not included. None of the participants had a lifetime history of neurological or severe systemic illness, head injury, or substance dependence.

The study was approved by the ethics committee of the Medical Faculty of Heidelberg University. Written informed consent was obtained from all participants in accordance with the Declaration of Helsinki.

### Clinical Assessment

Psychopathology was rated on the Brief Psychiatric Rating Scale [BPRS (24)], the Scales for the Assessment of Positive and Negative Symptoms [SAPS and SANS (25, 26)] and—to especially address apathetic symptoms—the Apathy Evaluation Scale [AES (27)]. This scale provides favorable psychometric properties in patients with various severe psychiatric disorders, including schizophrenia (19). For cognitive screening, the Mini-Mental State Examination (MMSE) was used (28).

Neurological soft signs were rated on the Heidelberg scale (4, 29), which comprises 16 items on 5 factors (motor coordination, sensory integration, complex motor tasks, right/left and spatial orientation, and hard signs). Ratings are given on a 0–3 point scale (no/slight/moderate/marked abnormality). A sufficient internal reliability (Cronbach’s alpha = 0.85–0.89), retest reliability (*r* = 0.80), and interrater reliability (*r* = 0.88) of the Heidelberg NSS scale were established in previous studies (3, 4).

Christina J. Herold, Marc M. Lässer, and Dusan Hirjak performed the clinical assessments, interrater agreements were ensured, possible ambiguities were discussed in periodic meetings. Blinding with respect to diagnostic group was not possible, given a patient sample with severe mental illness.

### Statistical Analysis

All statistical analyses were performed using SPSS version 23 (IBM SPSS Statistics). Group differences were analyzed by one-way (comparisons among patients) or two-way (comparisons across age and diagnosis groups) analysis of variance (ANOVA) or χ^2^-test, respectively. *Post hoc* tests were calculated *via* Bonferroni. Correlations between NSS and psychopathological symptoms were controlled for age and in a second step corrected for multiple tests using Bonferroni correction, which resulted in an adjusted significance level of *p* = 0.0009.

In addition, linear hierarchical regression analyses were performed to get information about variance explanation and contribution of the respective variables (according to their correlation with NSS) to NSS total score as dependent variable, while controlling for others (method: enter).

## Results

### Demographic and Clinical Characteristics

Patients and controls were divided into three age groups (18–29 years of age, 30–49 years of age, and +50 years of age), with the middle and older age group of the patients being institutionalized more often (*p* < 0.001).

Demographic and clinical characteristics of patients and healthy controls are given in Table 1. The healthy control group had—as to be expected—more years of education (*p* = 0.02) (patients: 95% CI [11.90, 13.00], controls: 95% CI [12.86, 14.37]). Moreover, a significant effect of “diagnosis” and “age group” could be observed for MMSE, with patients scoring significantly below the healthy controls (*p* < 0.001) (patients: 95% CI [26.22, 27.30], controls: 95% CI [28.44, 29.91]) and older subjects scoring well below younger subjects (*p* = 0.001). *Post hoc* tests revealed that young subjects differed significantly from old (*p* = 0.002) and middle-aged subjects (*p* = 0.023) (young: 95% CI [28.22, 30.10], middle: 95% CI [27.12, 28.63], and old: 95% CI [26.22, 27.55]). The interaction “diagnosis × age” failed significance level (*p* > 0.06).

**Table 1:** Demographic and clinical characteristics of patients and healthy controls.

	Patients (*n* = 90)			Healthy controls (*n* = 60)			Main effects and interaction, *F*-values_[df]_/χ^2^-values, effect size η^2^/φ		
		
	Young	Middle	Older	Young	Middle	Older	Diagnosis	Age group	Diagnosis × age
Sex m/f, *N* Male, %	17/14 54.8	23/10 69.7	15/11 57.7	5/5 50.0	10/7 58.8	18/15 54.5	χ^2^ = 0.554 *p* = 0.457 φ = 0.061	χ^2^ = 1.715 *p* = 0.424 φ = 0.107	N/A

Age, years	23.71 (3.18)	40.76 (5.85)	61.15 (7.46)	21.60 (3.06)	42.41 (5.30)	58.00 (7.08)	*F*_[1, 144]_= 1.312 *p* = 0.254 η^2^ = 0.009	*F*_[2, 144]_ = 408.966 ***p* < 0.001** η^2^ = 0.850	*F*_[2, 144]_ = 2.234 *p* = 0.111 η^2^ = 0.030

Education, years	12.42 (2.60)	12.26 (2.65)	12.67 (3.25)	14.20 (2.44)	13.12 (1.90)	13.52 (2.48)	*F*_[1, 144]_= 6.022 ***p* = 0.015** η^2^ = 0.040	*F*_[2, 144]_ = 0.561 *p* = 0.572 η^2^ = 0.008	*F*_[2, 144]_ = 0.365 *p* = 0.695 η^2^ = 0.005

MMSE sum score	28.68 (1.62)	26.52 (3.07)	25.08 (4.53)	29.60 (0.70)	29.24 (0.83)	28.70 (1.16)	*F*_[1, 144]_= 27.519 ***p* < 0.001** η^2^ = 0.160	*F*_[2, 144]_ = 7.743 ***p* = 0.001** η^2^ = 0.097	*F*_[2, 144]_ = 2.740 *p* = 0.068 η^2^ = 0.037

Medication, mg CPZ equivalents	726.00 (730.14)	730.42 (698.41)	577.42 (552.16)	N/A	N/A	N/A	N/A	*F*_[2, 86]_ = 0.466 *p* = 0.629 η^2^ = 0.011	N/A

Antipsychotic medication: AT, AT + T, T, no medication, *N* (%)	25/5/0/1 (80.6/16.1/0/3.2)	21/11/1/0 (63.6/33.3/3.0/0)	8/12/5/1 (30.8/46.2/19.2/3.8)	N/A	N/A	N/A	N/A	χ^2^ = 21.922 ***p* = 0.005**φ = 0.494	N/A

Additional antidepressive medication, *N* (%)	10 (32.3)	14 (42.4)	8 (30.8)	N/A	N/A	N/A	N/A	χ^2^ = 0.993 *p* = 0.609φ = 0.106	N/A

Additional benzodiazepines, *N* (%)	3 (9.7)	2 (6.1)	3 (11.5)	N/A	N/A	N/A	N/A	χ^2^ = 0.590 *p* = 0.745φ = 0.081	N/A

Illness duration, years	3.23 (3.47)	17.32 (9.88)	35.64 (8.64)	N/A	N/A	N/A	N/A	*F*_[2, 86]_ = 118.530 ***p* < 0.001** η^2^ = 0.734	N/A

Age at illness onset, years	20.70 (3.58)	23.55 (6.15)	25.40 (8.80)	N/A	N/A	N/A	N/A	*F*_[2, 83]_ = 3.836 ***p* = 0.026** η^2^ = 0.085	N/A

Institutionalized, *N* (%)	3 (9.7)	22 (66.7)	12 (46.2)	N/A	N/A	N/A	N/A	χ^2^ = 21.827 ***p* < 0.001**φ = 0.492	N/A

SAPS, sum score	21.27 (12.06)	13.42 (13.61)	15.46 (14.60)	N/A	N/A	N/A	N/A	*F*_[2, 86]_ = 2.785 *p* = 0.067 η^2^ = 0.061	N/A

SANS, sum score	19.83 (15.60)	28.27 (19.44)	23.77 (16.63)	N/A	N/A	N/A	N/A	*F*_[2, 86]_ = 1.854 *p* = 0.163 η^2^ = 0.041	N/A

BPRS sum score	41.53 (9.42)	39.82 (9.57)	34.85 (7.23)	N/A	N/A	N/A	N/A	*F*_[2, 86]_ = 4.186 ***p* = 0.018** η^2^ = 0.089	N/A

BPRS anxiety/depression	10.50 (4.40)	11.24 (5.03)	9.62 (4.62)	N/A	N/A	N/A	N/A	*F*_[2, 86]_ = 0.870 *p* = 0.423 η^2^ = 0.020	N/A

BPRS anergia	9.40 (4.26)	10.76 (5.11)	9.08 (4.20)	N/A	N/A	N/A	N/A	*F*_[2, 86]_ = 1.163 *p* = 0.318 η^2^ = 0.026	N/A

BPRS thought disturbance	10.80 (3.87)	8.46 (3.82)	7.85 (3.26)	N/A	N/A	N/A	N/A	*F*_[2, 86]_ = 5.207 ***p* = 0.007** η^2^ = 0.108	N/A

BPRS activity	4.40 (2.16)	4.94 (2.18)	4.23 (2.12)	N/A	N/A	N/A	N/A	*F*_[2, 86]_ = 0.896 *p* = 0.412 η^2^ = 0.020	N/A

BPRS hostility/suspiciousness	6.43 (3.44)	4.42 (2.11)	4.08 (1.60)	N/A	N/A	N/A	N/A	*F*_[2, 86]_ = 7.416 ***p* = 0.001** η^2^ = 0.147	N/A

AES, sum score	25.14 (12.94)	27.41 (10.80)	28.19 (13.18)	N/A	N/A	N/A	N/A	*F*_[2, 83]_ = 0.459 *p* = 0.633 η^2^ = 0.011	N/A

*Data are means (SDs), unless otherwise indicated*.

*AES, Apathy Evaluation Scale; AT, atypical antipsychotics; AT + T, atypical and typical antipsychotics; BPRS, Brief Psychiatric Rating Scale; CPZ, chlorpromazine; MMSE, Mini-Mental State Examination; N/A, not applicable; SANS, Scale for the Assessment of Negative Symptoms; SAPS, Scale for the Assessment of Positive Symptoms; T, typical antipsychotics*.

Within the patient group, the three age groups differed—necessarily—significantly with respect to duration of illness (*p* < 0.001), but not with respect to chlorpromazine (CPZ) equivalents (*p* > 0.60). While patients received mainly atypical antipsychotic medication (60%), a larger proportion of the elderly also received a combination of both, atypical and typical neuroleptics when compared with the younger age groups (*p* = 0.005). A minority of the patients received additional antidepressant (35.6%) or anxiolytic medication (8.9%). The three patient groups differed significantly in age of illness onset, with the youngest patients being younger at time of onset than the oldest patients (*p* = 0.024).

In case of the BPRS sum score, the young patients had significant higher scores than the old patients (*p* = 0.019), no other comparisons reached significance (*p* > 0.10). The BPRS subscales “thought disturbance” and “hostility/suspiciousness” showed significant higher scores for the young patients in contrast to both, middle-aged and old patients, respectively (0.001 < *p* < 0.05). The patient groups presented only minor, non-significant differences with respect to negative symptoms (BPRS Anergia, SANS) and apathy (AES).

### Group Comparisons—NSS

**Figure 1:**
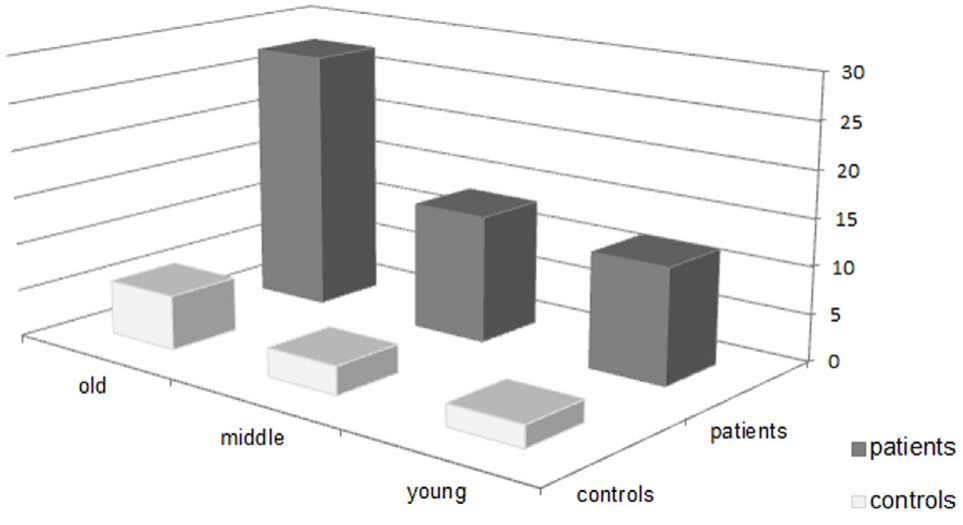
Neurological soft signs total scores of patients and healthy controls by age group.

The ANOVA calculated to compare NSS total scores between study groups yielded significant main effects for “diagnosis” and “age group,” with large to medium effect sizes. As demonstrated in Table 2 and Figure 1, patients had higher NSS scores than healthy controls (patients: 95% CI [15.76, 19.64], controls: 95% CI [1.03, 6.34]). Simultaneously, old subjects showed higher NSS scores than young subjects. *Post hoc* tests (*p* ≤ 0.009) revealed that the oldest subjects differed significantly from middle-aged and young subjects (young: 95% CI [3.86, 10.53], middle: 95% CI [5.48, 10.95], and old: 95% CI [14.26, 19.07]). The significant interaction “diagnosis × age” indicated different trajectories of age-related changes in patients and healthy controls, with small to medium effect sizes (patients young: 95% CI [8.80, 15.39], middle: 95% CI [10.23, 16.62], and old: 95% CI [23.98, 31.17]; controls young: 95% CI [−3.50, 8.10], middle: 95% CI [−1.45, 7.45], and old: 95% CI [2.57, 8.95]).

**Table 2:** Results of group comparisons on NSS.

	Patients (*n* = 90)			Healthy controls (*n* = 60)			Main effects and interaction, *F*-values_[df]_, effect size η^2^		
		
	Young	Middle	Older	Young	Middle	Older	Diagnosis	Age group	Diagnosis × age
NSS total score	12.10 (8.56)	13.42 (10.82)	27.58 (15.36)	2.30 (1.64)	3.00 (2.40)	5.76 (3.73)	*F*_[1, 144]_ = 70.753 ***p* < 0.001** η^2^ = 0.329	*F*_[2, 144]_ = 15.065 ***p* < 0.001** η^2^ = 0.173	*F*_[2, 144]_ = 6.468 ***p* = 0.002** η^2^ = 0.082

Motor coordination	5.65 (4.69)	4.74 (5.31)	10.81 (6.34)	0.50 (0.71)	1.29 (1.72)	2.33 (1.71)	*F*_[1, 142]_ = 53.307 ***p* < 0.001** η^2^ = 0.273	*F*_[2, 142]_ = 10.860 ***p* < 0.001** η^2^ = 0.133	*F*_[2, 142]_ = 4.437 ***p* = 0.014** η^2^ = 0.059

Sensory integration	1.32 (0.98)	2.29 (2.52)	4.19 (1.77)	0.40 (0.97)	0.35 (0.70)	1.24 (1.23)	*F*_[1, 142]_ = 45.386 ***p* < 0.001** η^2^ = 0.242	*F*_[2, 142]_ = 16.797 ***p* < 0.001** η^2^ = 0.191	*F*_[2, 142]_ = 4.162 ***p* = 0.018** η^2^ = 0.055

Complex motor tasks	2.29 (2.31)	2.19 (2.15)	4.46 (2.75)	0.80 (1.40)	0.65 (0.86)	1.39 (1.25)	*F*_[1, 142]_ = 31.946 ***p* < 0.001** η^2^ = 0.184	*F*_[2, 142]_ = 8.694 ***p* < 0.001** η^2^ = 0.109	*F*_[2, 142]_ = 2.434 *p* = 0.091 η^2^ = 0.033

Right/left and spatial orientation	1.52 (2.16)	3.19 (2.90)	5.46 (4.34)	0.40 (0.70)	0.35 (0.70)	0.36 (0.86)	*F*_[1, 142]_ = 44.336 ***p* < 0.001** η^2^ = 0.238	*F*_[2, 142]_ = 6.517 ***p* = 0.002** η^2^ = 0.084	*F*_[2, 142]_ = 6.693 ***p* = 0.002** η^2^ = 0.086

Hard signs	1.58 (1.43)	0.97 (1.43)	2.65 (2.61)	0.20 (0.63)	0.35 (0.70)	0.46 (0.94)	*F*_[1, 142]_ = 25.760 ***p* < 0.001** η^2^ = 0.154	*F*_[2, 142]_ = 4.664 ***p* = 0.011** η^2^ = 0.062	*F*_[2, 142]_ = 3.382 ***p* = 0.037** η^2^ = 0.045

*Data are means (SDs)*.

Similarly, analyses of NSS subscores demonstrated significant main effects for “diagnosis” (*F*_[5, 138]_ = 14.285, *p* < 0.001, η^2^ = 0.341) and “age group” (*F*_[10, 278]_ = 4.070, *p* < 0.001, η^2^ = 0.128) as well as the interaction “diagnosis × age” (*F*_[10, 278]_ = 2.375, *p* = 0.010, η^2^ = 0.079). Again, patients had *higher* NSS scores than healthy controls,^1^ and old subjects had higher NSS scores than young subjects *(p* ≤ 0.01) on all subscales. *Post hoc* tests showed a significant difference (*p* = 0.009) between old and middle-aged subjects in “motor coordination” (middle: 95% CI [1.73, 4.31], old: 95% CI [5.45, 7.69]). The subscore “sensory integration” differed significantly between old and middle (*p* = 0.009) and young subjects (*p* < 0.001) (young: 95% CI [0.29, 1.44], middle: 95% CI [0.85, 1.80], and old: 95% CI [2.30, 3.13]). In case of “complex motor tasks” the difference old vs. middle-aged subjects reached significance (*p* = 0.016) (middle: 95% CI [0.82, 2.02], old: 95% CI [2.41, 3.45]). “Right/left and spatial orientation” differed significantly between old and young subjects (*p* = 0.025) (young: 95% CI [0.05, 1.86], old: 95% CI [2.26, 3.57]). In case of “hard signs” *post hoc* tests did not survive the Bonferroni correction (*p* > 0.07).

The interaction “diagnosis × age” reached significance for all NSS subscales (*p* < 0.04), except for “complex motor tasks” (*p* = 0.09).^2^

These results were replicated with years of education as an additional covariate.

### Clinical Correlates of NSS

In the patients’ group, NSS total scores correlated significantly with duration of illness when age was controlled (*r* = 0.274, *p* = 0.011, df = 84). This was also the case for the subscales “motor coordination” and “complex motor tasks” of the Heidelberg NSS scale (*r* ≥ 0.25, *p* < 0.02, df = 84), see Table 3. Similar results were obtained when age and years of education were introduced as covariates. NSS total scores of the patients were not significantly correlated with CPZ equivalents (*p* > 0.60).

**Table 3:** Correlations between neurological soft signs (NSS) scales and psychopathology.

NSS scales/duration of illess^a^ BPRS subscales^a/^AES^b/^SAPS, SANS^a^	Duration of illness	BPRS anxiety/depression	BPRS anergia	BPRS thought disturbance	BPRS activity	BPRS hostility/suspiciousness	AES	SAPS	SANS
NSS sum score	0.27*	−0.06	0.05	0.27*	0.17	0.09	0.31**	0.35***	0.15
Motor coordination	0.25*	−0.01	−0.01	0.27*	0.15	0.15	0.24*	0.33**	0.03
Sensory integration	0.20	−0.07	0.10	0.27*	0.09	0.03	0.20	0.27*	0.15
Complex motor tasks	0.27*	−0.03	0.04	0.13	0.26*	−0.02	0.38***	0.27*	0.19
Right/left and spatial orientation	0.17	−0.12	0.11	0.08	0.20	−0.09	0.32**	0.15	0.22*
Hard signs	0.19	−0.09	−0.01	0.31**	−0.07	0.21	0.09	0.36***	0.12

*AES, Apathy Evaluation Scale; BPRS, Brief Psychiatric Rating Scale; SANS, Scale for the Assessment of Negative Symptoms; SAPS, Scale for the Assessment of Positive Symptoms*.

*^a^df = 84*.

*^b^df = 82*.

*p ≤ 0.05, **p ≤ 0.01, and ***p ≤ 0.001.

While a significant association between NSS total scores and BPRS sum scores (*p* > 0.10) did not arise, the BPRS subscale “thought disturbance” was significantly correlated (*r* = 0.267, *p* = 0.013, df = 84) with NSS total scores (Table 3). These results were replicated when age and duration of illness or years of education were taken into account.

Neurological soft signs total scores of the patients correlated significantly with SAPS sum scores (*r* = 0.348; *p* = 0.001; df = 84); all NSS subscales except for “orientation” were also significantly correlated with SAPS sum scores (0.36 ≥ *r* > 0.25; *p* < 0.02; df = 84). Very similar results were obtained with age and duration of illness or years of education as covariates.

The correlations of NSS with AES scores are summarized in Table 3. NSS total scores were significantly correlated with apathy (*r* = 0.309, *p* = 0.004, df = 82), this was also the case for the NSS subscales “motor coordination,” “complex motor tasks” and “orientation” (0.38 ≥ *r* > 0.20, *p* < 0.03, df = 82). Similar results were obtained when age and duration of illness or years of education were controlled. Moreover, these correlations could be reproduced when BPRS Anergia or SANS sum scores were introduced as additional covariates.

By contrast, a significant correlation between NSS total scores and negative symptoms as rated on the SANS and on the BPRS did not arise (*p* > 0.10).

When controlling the correlations for multiple testing using Bonferroni correction the association between NSS complex motor tasks and apathy remained significant, as it was the case for the associations between NSS total scores/hard signs and SAPS sum scores.

### Regression Analyses

The variables age, sex (0 female/1 male), education, CPZ equivalents, institutionalization (0—no/1—yes), and age at disease onset accounted for about 47% of the variance of NSS observed in the patients (Table 4). In the next step disease duration was added to the model. However, this variable was excluded due to multicollinearity, based on strong correlations between age and duration of illness (*r* = 0.91, *p* ≤ 0.001). In the next model, AES sum scores were introduced additionally, which resulted in a significant improvement of the model, accounting for 53% of the variance in total. The addition of SAPS sum scores and the BPRS subscale “thought disturbance” yielded a significant improvement of the model, which now explained 58% of the variance. Nonetheless, the beta coefficients of these two additional variables failed to reach significance level. The variables age, sex, education, institutionalization as well as age at disease onset showed significant effects, with negative contributions of all variables with the exception of age.

**Table 4:** Results of regression analyses (patients only).

	Variable	Standardized beta	*T*	*p*	*R*^2^	*F*_[df]_	*R*^2^ change	Sign. *F*change
**Model 1**					0.471	*F*_[6, 79]_ = 11.725***	0.471	11.725***
Step 1	Age	0.729	7.756	0.000				
	Sex	−0.157	−1.775	0.080				
	Education	−0.193	−2.017	0.047				
	Chlorpromazine (CPZ) equivalents	0.086	0.954	0.343				
	Institutionalization	−0.266	−2.807	0.006				
	Age at disease onset	−0.270	−2.881	0.005				

**Model 2**					0.530	*F*_[7, 75]_ = 12.060***	0.059	9.377**
Step 1	Age	0.671	7.295	0.000				
	Sex	−0.184	−2.132	0.036				
	Education	−0.051	−0.488	0.627				
	CPZ equivalents	−0.034	−0.357	0.722				
	Institutionalization	−0.304	−3.316	0.001				
	Age at disease onset	−0.240	−2.667	0.009				
Step 2	AES sum score	0.305	3.062	0.003				

**Model 3**					0.578	*F*_[9, 73]_ = 11.112***	0.049	4.196*
Step 1	Age	0.670	7.452	0.000				
	Sex	−0.202	−2.425	0.018				
	Education	−0.017	−0.168	0.867				
	CPZ equivalents	−0.108	−1.136	0.260				
	Institutionalization	−0.201	−2.113	0.038				
	Age at disease onset	−0.160	−1.748	0.085				
Step 2	AES sum score	0.352	3.573	0.001				
Step 3	SAPS sum score	0.182	1.356	0.179				
	Brief Psychiatric Rating Scale thought disturbance	0.093	0.674	0.502				

*p ≤ 0.05, **p ≤ 0.01, and ***p ≤ 0.001.

## Discussion

This study yielded two major findings: (i) while NSS increase with age in both, patients with schizophrenia and healthy controls, this effect is more pronounced in the patients group and (ii) increased NSS scores in patients with chronic schizophrenia are associated with duration of illness, positive symptoms/thought disorder, and apathy.

Neurological soft signs were found to be significantly elevated in patients with chronic schizophrenia when compared with healthy controls. The latter showed rather low levels of NSS; their values were comparable to those of other studies (30, 31) also using the Heidelberg Scale for NSS assessment (4, 29).

That NSS are increased in schizophrenia is demonstrated in a wealth of studies including recent meta-analyses (5, 32). By contrast, evidence with respect to age effects on NSS is rather scarce. Chan et al. (33) demonstrated significant age-differences of NSS with a U-shaped pattern in healthy subjects who were between 14 and 62 years of age. Both adolescents and those over 50 years of age showed significantly higher scores than subjects in between. The increase of NSS scores in adulthood parallels the age effect found in this study, which involved patients with chronic schizophrenia and healthy controls aged between 18 and 82 years. Urbanowitsch et al. (34) found only minor non-significant differences between two large birth cohorts of otherwise healthy subjects recruited from the general population. Subjects were examined in their mid 50s and mid 70s after being carefully screened for health by history taking, clinical examination including laboratory testing and neuropsychological investigation. This is of primary importance as cognitive losses typically involve increased NSS scores (34, 35). This interpretation is further supported by the significantly reduced MMSE scores in our patients that also deteriorate with age, although the interaction “diagnosis × age” did not reach significance level. Taken together, our findings suggest that NSS increase from early adulthood to midlife, an effect that is more pronounced in the patients group.

Similarly to our results Chen et al. (36) found NSS to deteriorate in a sample of 43 patients with chronic schizophrenia in their early 50s over a 3-year period, whereas pyramidal and extrapyramidal signs and signs of dyskinesia appeared to be rather stable and changes in symptoms and medication were only minimal. By contrast, evidence for the stability of neurological dysfunction came from a former publication of this workgroup (37). 204 patients between 16 and 65 years of age were divided into 6 groups according to their duration of illness (<5, 6–10, 11–15, 16–20, 20–25, and >25 years). While subgroups with a longer duration of illness had significantly higher NSS scores, this effect disappeared when age and education were controlled for, with the exception of the subscale “motor coordination.” By contrast, our results were confirmed when years of education were partialed out. Hence, one may hypothesize that high NSS scores in the chronically ill may also be associated with lower educational levels (see also regression analyses of patients, model 1); a hypothesis that conforms to the fact that the disease often prevents patients from achieving a higher school and/or professional education, which is generally considered as a surrogate of cognitive reserve. That cognitive reserve also involves an ameliorating effect on NSS was demonstrated in the population based study cited above (34) for both, otherwise healthy controls and patients with mild cognitive impairment/Alzheimer’s disease.

In previous studies on patients with chronic schizophrenia NSS were significantly correlated with the severity and persistence of psychopathological symptoms (4, 6, 38, 39). While associations between NSS and BPRS scores were replicated in this study, these associations do not seem to be consistent across different samples (4, 8, 39, 40). In their review on NSS and psychopathology in schizophrenia, Tosato and Dazzan (41) concluded that NSS associated with negative symptoms may remain stable over time, thus characterizing a subgroup with poor illness course/outcome; by contrast, NSS associated with positive symptoms may improve with decreasing symptoms. The correlations between NSS and SAPS/BPRS “thought disturbance” in our study may therefore correspond to the fluctuation of positive symptoms during the course of the disorder. This interpretation is indirectly facilitated by the clinical observation of a decline of positive symptoms with aging.

Although Tosato and Dazzan (41) summarized a wealth of studies, trials which focused on older patients with chronic schizophrenia were obviously not available at this time. In this study, significant associations with NSS could only be demonstrated for apathetic but not negative symptoms. From a psychopathological standpoint, apathy is a much broader concept than negative symptoms since it can arise in various severe neuropsychiatric disorders including schizophrenia, mild cognitive impairment and Alzheimer’s disease (19, 20). In old patients with dementia, apathetic symptoms are highly correlated with cognitive deficits, which can also be demonstrated in old patients with chronic schizophrenia (13, 20) as already conceptualized in Kraepelin’s original hypothesis of dementia praecox (18). However, possible associations between NSS and neuropsychological performance in patients with chronic schizophrenia are addressed in detail in another publication of our group (42).

Sex seemed to influence—at least partly—NSS scores, as reflected by significant contributions of this variable in some of the regression analyses. However, the sex distribution of our groups was somewhat unequal, as indicated by 70% males in the middle-aged patients. An influence of sex was not confirmed by Bartko et al. (43) in a sample of patients with chronic schizophrenia. Heinrichs and Buchanan (44) described rather mixed results while Chan and colleagues (32) were not able to address this issue in their recent meta-analysis due to missing data in the primary studies. Furthermore, higher age at disease onset may be a protective factor according to the results of the regression analyses, which conforms to the general observation that a rather late onset enables patients to reach at least their graduation or professional education in contrast to patients with a very early onset, thus contributing to cognitive reserve. Institutionalization also had a favorable influence on NSS total scores, a finding which seems to be surprising at first glance. However, non-institutionalized patients who received clinical treatment at time of examination had higher NSS scores because NSS fluctuate due to the negative impact of symptom severity as described earlier.

When interpreting our results of elevated NSS with increasing age and chronicity of schizophrenia, the potential side effects of neuroleptic medication have to be considered as a potential confounder.

As in this study, Chen et al. (37) did not find a significant correlation between antipsychotic dosage and NSS scores. While we did not specifically address extrapyramidal symptoms, Jahn et al. (39) described that NSS were not significantly different between patients receiving atypical vs. conventional neuroleptics, concluding that NSS in schizophrenia are relatively independent of neuroleptic side effects, but instead were associated with severity and persistence of psychopathological symptoms. Studies with neuroleptic-naïve patients and/or with first-episode of schizophrenia have demonstrated that NSS are present before neuroleptic exposure [overview (45, 46)]. That NSS are rather independent of antipsychotic medication is also supported by the findings of elevated NSS in high risk subjects (47) or relatives of people with schizophrenia (48).

Furthermore, previous clinical reports described higher NSS scores in (drug-naïve) patients decreasing under antipsychotic treatment (4, 40, 49, 50). Along with this, Chen and colleagues (51) showed elevated levels of NSS in patients with first-episode schizophrenia including medication-naïve subjects, while a shorter duration of untreated psychosis had a positive effect on NSS scores.

Another possible limitation of this study is a selection bias concerning the high percentages of institutionalized patients in our older group. However, patients with a long duration of illness and a poor outcome are typically unable to fully care for themselves due to a poor social and cognitive functioning.

This study did not involve repeated examination of patients in the clinical course. If confirmed in a longitudinal study, the increase of NSS scores with both, age and chronicity of the disease had the potential to facilitate our understanding of schizophrenia and to better predict its clinical course.

## Ethics Statement

The study was approved by the ethics committee of the Medical Faculty of Heidelberg University. Written informed consent was obtained from all participants in accordance with the Declaration of Helsinki.

## Author Contributions

CH performed data collection and statistical analyses and wrote the manuscript. ML performed data collection and supported interpretation of data. PT, DH, and US were involved in designing the study and supported data collection. JS designed the study, supervised clinical assessments, contributed to the interpretation of the results, and supported critical revision of the manuscript. All the authors participated in critical revising and final approval of the manuscript and agreed to be accountable for all aspects of the work.

## Conflict of Interest Statement

The authors declare that the research was conducted in the absence of any commercial or financial relationships that could be construed as a potential conflict of interest.

## Footnotes

^1^Patients: 95% CI motor coordination [6.15, 7.98], 95% CI sensory integration [2.26, 2.94], 95% CI complex motor tasks [2.56, 3.40], 95% CI right/left and spatial orientation [2.86, 3.92], and 95% CI hard signs [1.41, 2.06]; controls: 95% CI motor coordination [0.14, 2.62], 95% CI sensory integration [0.21, 1.12], 95% CI complex motor tasks [0.37, 1.52], 95% CI right/left and spatial orientation [−0.35, 1.09], and 95% CI hard signs [−0.10, 0.77].

^2^Motor coordination patients young: 95% CI [4.11, 7.18], middle: 95% CI [3.21, 6.28], and old: 95% CI [9.13, 12.49]; controls young: 95% CI [−2.20, 3.20], middle: 95% CI [−0.78, 3.37], and old: 95% CI [0.85, 3.82]. Sensory integration patients young: 95% CI [0.76, 1.89], middle: 95% CI [1.72, 2.86], and old: 95% CI [3.57, 4.81]; controls young: 95% CI [−0.60, 1.40], middle: 95% CI [−0.41, 1.12], and old: 95% CI [0.69, 1.79]. Right/left and spatial orientation patients young: 95% CI [0.62, 2.41], middle: 95% CI [2.30, 4.09], and old: 95% CI [4.49, 6.44]; controls young: 95% CI [−1.17, 1.97], middle: 95% CI [−0.85, 1.56], and old: 95% CI [−0.50, 1.23]. Hard signs patients young: 95% CI [1.04, 2.12], middle: 95% CI [0.43, 1.51], and old: 95% CI [2.06, 3.25]; controls young: 95% CI [−0.76, 1.16], middle: 95% CI [−0.38, 1.09], and old: 95% CI [−0.07, 0.98].
